# Easy read and accessible information for people with intellectual disabilities: Is it worth it? A meta‐narrative literature review

**DOI:** 10.1111/hex.12520

**Published:** 2016-11-16

**Authors:** Deborah Chinn, Claire Homeyard

**Affiliations:** ^1^ Florence Nightingale Faculty of Nursing and Midwifery King's College London London UK

**Keywords:** accessibility, easy read, health information, intellectual disabilities, meta‐narrative review

## Abstract

**Background:**

The proliferation of “accessible information” for people with intellectual disabilities in UK health care has accelerated in recent years, underpinned by policy guidance alongside the recent introduction of mandatory standards. However, questions have been raised as to the impact of such resources as a means of enhancing involvement in health care and addressing health inequalities.

**Objective:**

To review and synthesize the evidence from different approaches used to evaluate the impact of accessible information for people with intellectual disabilities using a meta‐narrative approach.

**Search strategy:**

Literature searches were iterative and incorporated formal databases, grey literature and hand searches alongside more intuitive and opportunistic methods.

**Inclusion criteria:**

Included English language papers published before December 2015 described the design and evaluation of written information adapted for adults with intellectual disabilities.

**Data extraction and synthesis:**

We organized the papers into five groups according to similarity in authors’ writing styles and presentation, epistemology and theoretical foundations, aims and methodologies, professional and organizational identities.

**Main results:**

The 42 included papers in the five groupings occupied diverse positions on (i) public communication vs individualized materials, (ii) literacy as decontextualized skills vs social practices and (iii) the expertise of patients vs professionals. There was limited evidence for the impact of accessible health information, notwithstanding the potential benefits associated with their creation.

**Conclusions:**

Individually tailored information is more likely to meet personalized health information needs for people with intellectual disabilities. The emergence of different social formations in the creation of accessible information has potential for advancing engagement of diverse groups.

## 1 INTRODUCTION

The provision of “accessible information” to people with intellectual disabilities is often characterized as the answer to some “wicked” (persistent, resistant to solution) problems^1^ relating to equal access to health and well‐being. Policy documents and statements by politicians, practitioners, researchers and self‐advocates suggest that accessible information will allow people with intellectual disabilities to become more self‐determining, minimize health inequalities, promote active citizenship and bring about the empowerment of a social group whose voice is often excluded and ignored.^2^, ^3^

Consequently, the availability of accessible information has proliferated greatly in recent years, especially in the health field. There are a number of guidance documents available that supply instructions for creating accessible resources^4^, ^5^ and an ever widening range of information resources have been published in an accessible information format (a recent UK web search turned up 24 different resources relating to blood tests alone). The requirement for service providers to make “reasonable adjustments” to their provision, including information they offer about their services, is now enshrined in the 2010 Equalities Act.^6^ The establishment of the NHS England Accessible Information Standard^7^ is a recent attempt to operationalize the adjustments that are expected from all health and social care agencies.

There have been some more sceptical voices, raising questions about the real impact of such resources and whether they are in fact fit for purpose, conveying all the necessary information in a clear and simple way.^8^, ^9^ Some have queried whether the import of accessible information is more symbolic as a marker of ideological commitment to inclusion, rather than a practical means of enhancing the knowledge of people with intellectual disabilities.^8^, ^10^ Others have highlighted the risk that accessible information will be handed to people with intellectual disabilities without appropriate support or attention to their individual communication needs.^11^

To date, there is little systematically reviewed evidence that supports this policy‐level commitment to adapting health information for people with intellectual disabilities by confirming its effectiveness. This review is therefore an attempt to fill this gap and moreover to include a wide range of perspectives and approaches to exploring the value of accessible information.

## 2 METHODOLOGY

Greenhalgh and colleagues^12^, ^13^ suggest “meta‐narrative” as an approach to literature reviewing that does not seek to iron out major theoretical and methodological differences between studies, but collects similar studies into defined research traditions and paradigms. Each of these traditions brings to bear its own epistemology and methodology in addressing the research question. The reviewers’ job is to highlight the insights from each tradition, comment on the agreements and disagreements between them compare and come up with higher order themes that encompass these. This orientation is explicitly constructivist; the focus is on systems of meaning making associated with different paradigms, rather than determining any one underlying truth.

**Figure 1:**
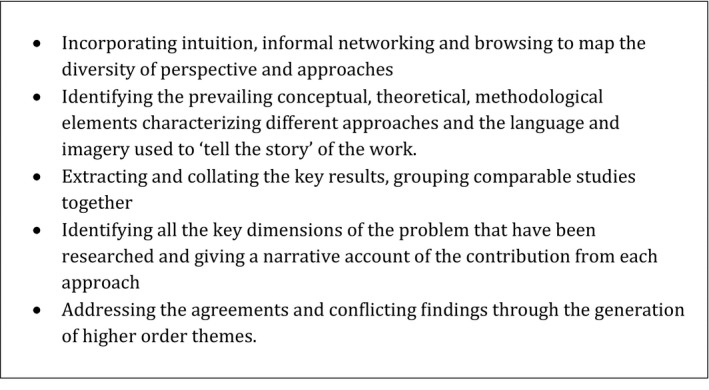
Steps for undertaking meta‐narrative review (from Greenhalgh et al. 12)

More recently, the principles of meta‐narrative review have been used in smaller scale studies^14^, ^15^ with a more focused scope than the original, wide ranging reviews. In similar vein, we adopted the key strategies of meta‐narrative review (see Figure 1), keeping in mind the six guiding principles of this approach (pragmatism, pluralism, historicity, contestation, reflexivity and peer review) and using recent guidance^16^, ^17^ as a benchmark to evaluate the quality of our study design, execution and writing up. Our research questions were:

1 How has the impact of accessible health information for people with intellectual disabilities been evaluated? Are there different groupings that might represent “bodies of knowledge” relating to this field?
2 In each of these groupings
  ○ What are the underpinning concepts, theories and methodological approaches?
  ○ Are foundational and high‐quality studies identified?
  ○ What are the key empirical findings and conclusions?
3 Comparing the different groupings,
  ○ Can commonalities be found between the assumptions, approaches, findings and conclusions of the different groups?
  ○ How do agreements and disagreements among the groupings suggest higher order insights?
4 What further research is indicated?

### 2.1 Definitions

The definition of “accessible information” for people with intellectual disabilities raised challenges. There is no single generally accepted conceptualization, which means it is often ill‐defined within contemporary policy documents. Within health‐care settings, understandings are skewed towards easy‐to‐read resources,^5^ although others have insisted that the mode of delivery of information^18^ or the involvement of people with intellectual disabilities in creating accessible information resources^2^ is key.

For instance, “easy read” is a term that has come into common use in the UK to describe information designed specifically for people with intellectual disabilities as a group with particular literacy needs. It has supplanted other terms (“easy to read,” “easier information”) and is found on many adapted public documents. This term is not so common in other English‐speaking countries, although is gaining ground in Australasia.

## 3 METHODS

### 3.1 Scoping

The initial scoping phase of the review involved reflecting on our prior knowledge as researchers and clinicians. We contacted others engaged in similar work, tracked citations from the reference lists of guidance documents and opinion pieces and embraced serendipitous discoveries of different areas of work. A combination of Medical Sub‐Headings (MeSH) terms supplemented with free‐text words relating to “accessible information,” “easy read”/”easy to read,” and terms for intellectual disabilities was drawn up to identify potentially relevant literature in searchable bibliographic databases.

### 3.2 Searching

We conducted a search in December 2015 Maternity and Infant Care (MIC), United States National Library of Medicine's bibliographic database (MEDLINE), American Psychological Association (PsycINFO), Excerpta Medica Database (EMBASE), Health Management Information Consortium (HMIC) and Cumulative Index to Nursing & Allied Health Literature (CINHAL).

Next, an iterative process was initiated whereby we identified additional references by hand searching the reference lists and citations of relevant papers. We included “grey literature” such as reports published by public bodies found through Internet searching.

### 3.3 Study selection

**Figure 2:**
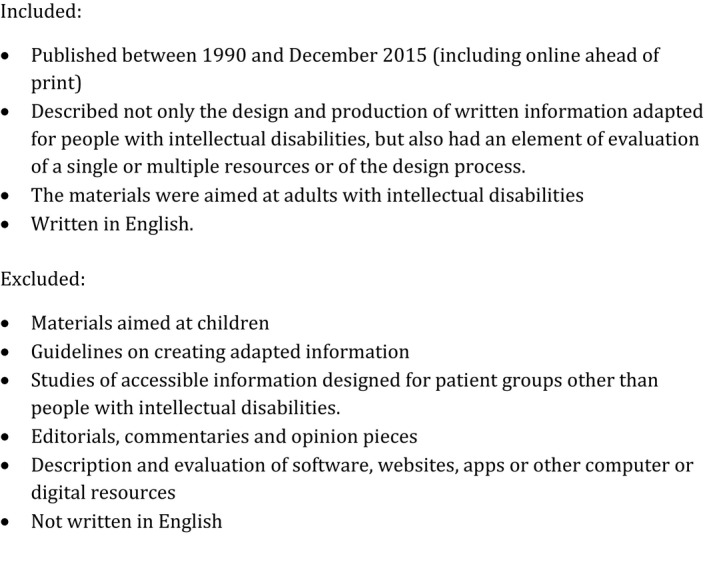
Inclusion and exclusion criteria

Study titles and abstracts were screened for eligibility, allowing for discussion to resolve any uncertainties. For studies not excluded on title and abstract, we obtained the full paper and assessed it in more detail. We established inclusion and exclusion criteria in order to come up with a more manageable number of papers, whilst avoiding an overly restrictive limitation of the studies to be included (Figure 2). Although our primary interest was in health information, we did not restrict our search to that field; a few papers were included that looked at information relating to voting,^19^ consent to participation in research^20^ or the criminal justice system.^21^ We did not include video or digital resources. We recognize that these formats offer many exciting possibilities for people with intellectual disabilities; however, their creation and use require access to relatively specialist and expensive resources, compared to print media or audio recordings.

### 3.4 Data extraction

We tabulated key bibliographic features of the studies. Reading, rereading and discussing our selected studies with an eye on this table helped us to identify groupings of papers where there were commonalities in styles of writing and presentation; aims, methodologies and study design; professional or organizational identities and ideological affiliations. This was not a one‐off process; our categorization underwent considerable adjustment and refinement. We did not find a great deal of cross‐referencing between studies and struggled to identify “seminal” or foundational studies that received most citations and themselves stimulated development of what might be identified as a “research tradition.” In some cases, we went further than the authors themselves in associating them with wider research fields or epistemological frameworks as these aspects of the papers were largely implicit.

Inductive coding using NVivo 11 (QSR, 2015) of one group of papers led us to create a coding framework that we then applied to the other groupings. This allowed us to contrast and compare across the different groupings. To enhance consistency of data extraction, we coded the same two papers individually and then compared the data extracted and code headings. Following discussion and agreement on the code headings, we divided the remaining papers and coded them.

### 3.5 Quality appraisal

Is often not appropriate for a meta‐narrative review to apply the same quality criteria to all the studies under review. Where distinct approaches to the research topic have been identified, Wong et al.^16^ recommend that “studies in these separate traditions should be appraised using the quality criteria that a competent peer‐reviewer in that tradition would choose to use (p19)” rather than following a single pre‐determined protocol to evaluate quality. Therefore, considerations of study quality are addressed within the summaries of each research grouping in the results section below. Nevertheless, it is important to note that only the papers published in practitioner or academic journals, as distinct from grey literature and reports, were subjected to a transparent peer‐review process.

## 4 RESULTS

**Figure 3:**
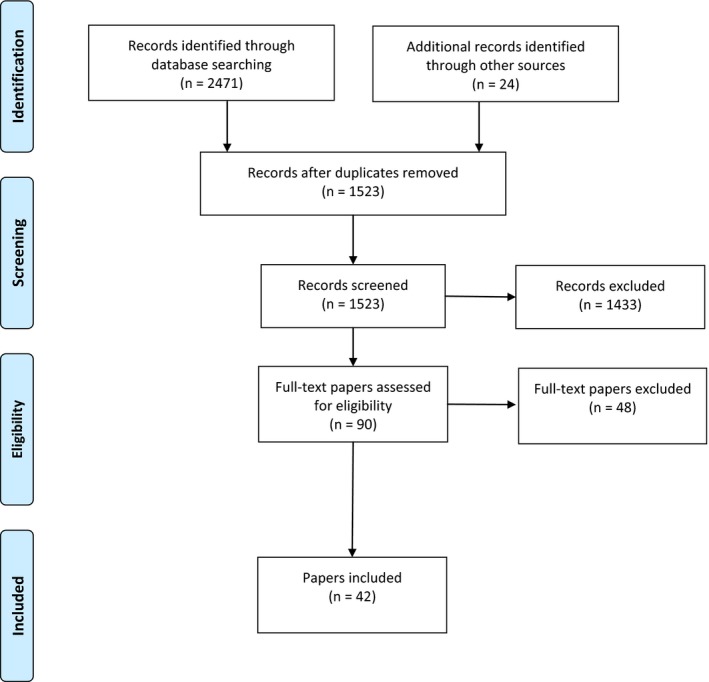
Systematic review process. [Colour figure can be viewed at wileyonlinelibrary.com ]

Forty‐two papers were included in the review (see Figure 3), which we collated into 5 groups (Table 1). The most recent print versions of papers are listed. In this section, we present accounts of each group before offering a synthesis of findings and inferences across the groups.

**Table 1:** Grouping studies for meta‐narrative review

	Description	Key characteristics	Studies included
Group 1	Practitioner Accounts	Authored mainly by clinicians. Description of development and mainly qualitative audience testing of own resources.	Aman et al. (2007) Project MED: Effects of a Medication EDucation booklet series for individuals with intellectual disabilities *Intellectual and Developmental Disabilities*. 45 (1), 33–45.
			Dawson (2011) How to make information on health care accessible to all. Learning Disability Practice. 14 (4), 23‐25.
			Gaudion et al. (2013) Mothers with a learning disability: access, information provision and ongoing engagement in antenatal care. Available at http://thepolyannaproject.org.uk/resources/polyanna_project-mothers_with_a_learning_disability.pdf
			Gilbert et al. (2007) Supporting people with intellectual disability in the cancer journey: The ‘living with cancer’ communication pack. *European Journal of Oncology Nursing*. 11(4), 357–61.
			Howieson & Clarke (2013) Ensuring service users can access crucial information*, Learning and Disability Practice*. 16(1), 22‐25.
			Kelly (2011) Diabetes and Me: Learning disabilities and diabetes. *Journal of Diabetes Nursing*. 15 (8), 308‐312.
			King (2011) Clear information can cut inequalities in learning disabilities. *Nursing Times*. 107 (4), 22–24.
			Parsons, & Sherwood (2016). A pilot evaluation of using symbol‐based information in police custody. *British Journal of Learning Disabilities*. 44(3), 212‐224.
			Porter et al. (2012) Developing the pregnancy support pack for people who have a learning disability. *British Journal of Learning Disabilities,* 40(4), 310‐317.
			Poynor (2003) Being ‘breast aware’. *Learning Disability Practice*, 6 (4), 10‐14.
			Russell (2006) Developing health resources with the help of people with Down Syndrome, *Learning Disability Practice* 9 (4), 16–18.
Group 2	People with intellectual disabilities as resource evaluators	Authors were from disability studies and self‐advocacy backgrounds. Foregrounded opinions of people with intellectual disabilities.	Clark (2002) Accessible Health Information: Project Report. Liverpool: Liverpool Central Primary Care Trust.
			Codling & Macdonald (2008) User‐friendly information: does it convey what it intends? *Learning Disability Practice,* 11(1), 12‐17.
			Dowds (2011) Evaluation of CHANGE resources to support the information needs of parents with learning disabilities with professionals. Available at http://www.healthscotland.com/.
			Essex County Council (2015) Annual Health checks: a report on Easy Read Information. Available at https://www.improvinghealthandlives.org.uk/adjustments/?adjustment=347
			Ledger & Shufflebotham (2003) Easy guide to physical interventions for people with learning disabilities, their carers and supporters. British Journal of Learning Disabilities. 31, 103‐105.
			Lewis et al. (2011) Evaluation with expectant and new parents with children from pregnancy to age 5 y of CHANGE resources to support parents with learning disabilities. Available at https://www.choiceforum.org/docs/enewpr.pdf.
			Marriott & Tarleton (2008) Finding the Right Help. Bristol: Norah Fry Research Centre.
			Turnpenny et al. (2015) Developing an Easy Read version of the Adult Social Care Outcomes Toolkit (ASCOT). 2015 Available at https://kar.kent.ac.uk/49040/1/4907.pdf
Group 3	People with intellectual disabilities reflecting on processes of creation of resources	Authored/co‐authored by people with intellectual disabilities, aligned with principles of inclusive and participatory research	Can you understand it group (2014) Oxleas ‘Can you understand it?’ group. *Advances in Mental Health and Intellectual Disabilities*. 8(4), 268–270.
			Goodwin et al. (2015) Easy Information about research: getting the message out to people with learning disabilities. *British Journal of Learning Disabilities*,* 43*(2), 93–99.
			Tuffrey‐Wijne & Bernal (2003) ‘Getting on’ with cancer. Learning Disability Practice. 6(5), 10‐15
			Wyre Forest Self Advocacy & Tarleton (2005) Writing it ourselves. *British Journal of Learning Disabilities*. 33(2), 65–9.
Group 4	Observations of accessible information in use	Addressed the question of how accessible information was used to address real‐life issues. Interest in interaction context where accessible information might be used.	Dodd & Brunker (1999) ‘Feeling poorly’: report of a pilot study aimed to increase the ability of people with learning disabilities to understand and communicate about physical illness. *British Journal of Learning Disabilities*. 27(1), 10–15.
			Jones et al. (2006) Meeting the cancer information needs of people with learning disabilities: experiences of paid carers. *British Journal of Learning Disabilities*. 35(1), 12–18.
			Mander (2016) aAn investigation of the delivery of health‐related accessible information for adults with learning disabilities. *Tizard Learning Disability Review*, 21(1), 15‐23.
			Mander & Rigby (2014) Obtaining patient feedback for doctors’ revalidation using accessible resources. Available from at http://www.solent.nhs.uk/_store/documents/accessible_patient_feedback_report.pdf.
			Macer & Fox (2010) Using a communication tool to help clients express their health concerns. *Learning Disability Practice*. 13(9), 22–4.
			Oldreive & Waight (2013) Enabling access to information by people with learning disabilities. *Tizard Learning Disability Review*. 18 (1), 5–15.
			Tuffrey‐Wijne et al. (2006) People with intellectual disabilities and their need for cancer information. *European Journal of Oncology Nursing*. 10(2), 106‐16.
Group 5	Evaluation of the effects of accessible information	Use of experimental paradigms under relatively controlled conditions to investigate whether accessible information was easier to read and understand for people with intellectual disabilities.	Cardone (1999) Exploring the use of question methods: pictures do not always help people with learning disabilities*. The British Journal of Development Disabilities*. 45(89), 93–98.
			Dye et al. (2006) Capacity to consent to participate in research–a recontextualization. *British Journal of Learning Disabilities*. 32(3), 144–150.
			Fajardo et al. (2012) Easy‐to‐read texts for students with intellectual disability: linguistic factors affecting comprehension. *Journal of Applied Research in Intellectual Disabilities*. 27(3), 212‐225.
			Feldman & Case (1997). The effectiveness of audiovisual self‐instructional materials in teaching child‐care skills to parents with intellectual disabilities. *Journal of Behavioral Education*, 7(2), 235‐257.
			Huenerfauth et al. (2009) Comparing evaluation techniques for text readability software for adults with intellectual disabilities. In: *Proceedings of The 11th International ACM SIGACCESS Conference on Computers and Accessibility* ACM; 2009, p. 3–10. Available at http://dl.acm.org/citation.cfm?id=1639646
			Hurtado et al. (2013) Is Easy Read information really easier to read? *Journal of Intellectual Disability Research*. 58(9), 822‐829.
			Jones et al. (2007) Symbols can improve the reading comprehension of adults with learning disabilities. *Journal of Intellectual Disability Research*. 51(7):545–550.
			Murphy & Cameron (2008) The effectiveness of talking mats® with people with intellectual disability. *British Journal of Learning Disabilities*, 36, 232‐241.
			Poncelas & Murphy (2007) Accessible information for people with intellectual disabilities: Do symbols really help? *Journal of Applied Research in Intellectual Disabilities,* 20(5), 466‐474.
			Strydom et al. (2001) Patient information leaflets for people with learning disabilities who take psychiatric medication. *British Journal of Learning Disabilities*, 29(2), 72‐76.
			Strydom & Hall (2001) Randomized trial of psychotropic medication information leaflets for people with intellectual disability. *Journal of Intellectual Disability Research*, 45 (2), 146‐151.
			Yaneva (2015) Easy‐read documents as a gold standard for evaluation of text simplification output. In: *ACL Student Research Workshop*. Available at http://www.anthology.aclweb.org/R/R15/R15-2.pdf#page=38

a Online ahead of print was available from November 2015.

### 4.1 Group 1: Practitioner accounts

The authors of these articles were mainly clinicians who created their own resources for distribution or use by a specified group of people with intellectual disabilities. These articles were generally published in journals accessed by practitioners that provided opportunities for authors to share information and experiences and publicize their work and raise the profile of the service they worked in, rather than advance theory or methodology. The 12 papers we placed in this group on the whole provide more detail on the processes involved in creating accessible resources, with less information about the evaluation of their impact.

Authors orientated at the beginning of their papers to the “rights agenda,” the legislative context of equalities legislation, as well as health inequalities experienced by people with intellectual disabilities. Nevertheless, the authors generally took for granted a traditional patient education framework^22^ in which expert knowledge and advice is primary. In this model, the role of health education is to clarify and disseminate the message for uptake by patients, who are assumed to be lacking in knowledge and skills necessary to maintain health. Justifications given by authors for undertaking adaptations highlighted the knowledge and communicative deficits of people with intellectual disabilities.^23^, ^24^

In addition, the professional and organizational needs of practitioners and service providers were foregrounded. Practitioners as well as people with intellectual disabilities were featured as producers and recipients of accessible information that was proposed as a component of improving service quality, particularly the goal of improving the capabilities of mainstream services to work effectively with people with intellectual disabilities. Moreover, authors furnished examples of specialist intellectual disability staff and mainstream health staff coming together to work on resource production.^23^, ^25^, ^26^ Some authors also made reference to their adapted materials being helpful for a range of service users who might struggle with reading English, a further justification for service time and energy to be put into creating and disseminating these resources.^21^, ^23^, ^27^

Evaluation was conducted mainly via focus groups of people with intellectual disabilities or staff, interviews or through monitoring uptake of the resource. Analysis of responses was mainly qualitative (only Aman et al. 27 presented statistical analysis of responses) with, in some cases, sparse details about specific methods of collecting or analysing data.

These papers present an overwhelmingly positive view of the resources under consideration, although the lack of detail in some accounts (simple statements to the effect of “the material was well received”^28^) does beg the question of how far opportunities were offered to elicit more critical responses. Moreover, there may be a risk of publication bias, since initiatives that met with more lukewarm responses are less likely to be written up, especially by busy practitioners. Authors also told positive stories of resources being taken up in large numbers by health and social care organizations as indications of the success of the accessible information project.^25^, ^29^

### 4.2 Group 2: People with intellectual disabilities as resource evaluators

These authors of the nine papers in this group described projects where the opinions of people with intellectual disabilities were foregrounded as reviewers of accessible information resources (one or many) that had been created elsewhere. In four cases, authors were part of independent research teams who had been commissioned to review a specific resource.^21^, ^30^, ^31^, ^32^ Participants were in many cases associated with self‐advocacy organizations 31, 33 or those that worked with people with intellectual disabilities as coresearchers.^34^, ^35^

The authors of these papers were more likely to align explicitly with the social model of disability.^36^ The importance of people with intellectual disabilities taking an equal role in designing accessible resources and also having control over when and how the resources were used was emphasized. Authors drew attention to power imbalances between people with intellectual disabilities and others (carers and paid staff). They suggest that staff and supporters themselves can present barriers to disabled people in terms of distribution of resources and their use to promote free and unbiased decision making by people with intellectual disabilities.^32^

The findings of this group were more ambivalent than Group 1. Many participants in these studies were appreciative of efforts to make written information more accessible. However, in most of these papers, limitations in the accessible information were highlighted, particularly ambiguous visual images^32^, ^33^ and wordings,^37^ having too much information, being too difficult to read, even for “competent readers”.^37^, ^38^ Some authors pointed out how simplifying a visual image or written information made its meaning less, rather than more clear.

Perhaps because of their background in disability studies and self‐advocacy, these authors offered a more complex understanding of access for people with intellectual disabilities^39^ that encompassed not only the existence of adapted resources, but also their distribution and availability. Many of their participants mentioned problems in getting hold of resources^30^, ^31^, ^36^, ^38^; these constituted further barriers to access and might be caused by funding for a resource drying up 34, 35 or professionals not handing them on to people with intellectual disabilities.^30^

### 4.3 Group 3: People with intellectual disabilities reflecting on process of creating resources

The three studies in this area were papers authored or co‐authored by people with intellectual disabilities, or referred to work authored by someone with intellectual disabilities. They appeared in British specialist intellectual disability journals that are welcoming of papers in easier to read formats,^40^ and all included illustrations. The stated aim of these studies was to highlight the capabilities of people with intellectual disabilities as producers of accessible information. In many ways, they depart from what is usually considered acceptable as a research report, with little reference to background academic literature or impersonal appraisal of data. Their value is judged to reside in their close alignment with the principles of inclusive/participatory research as reflecting the agency and voice of people with intellectual disabilities.^41^

There is an awareness of how creation and use of written information engage not only the cognitive aspects of the self, but also sociality and emotion. The self‐advocate authors mentioned how their role/job gives them social identity and enhances social bonds and self‐esteem.^42^, ^43^ Moreover, their involvement allows them to operate within what Glynos & Speed call “a regime of recognition” made available through coproduction models^44^ that publicly foregrounds the capabilities, rather than deficits of disabled people.

### 4.4 Group 4: Observations of accessible information in use

These qualitative studies involved reports or observations of people with intellectual disabilities making use of adapted information resources in relatively naturalistic environments or to address real‐life issues. All of the lead authors were learning disability specialist practitioners from allied health professions.

The papers described ways in which the adaptation was personalized for the individual with intellectual disabilities. The role of supporters and the importance of their expertise in facilitating use of accessible information were highlighted. The authors’ concept of “informational accessibility” did not begin and end with simplifying a specific text; they emphasized the importance of prior assessment of individual communicative capacities and needs^45^ and of the mode of delivery of adapted information.^18^

Their conclusions were mixed. In contexts where the information under discussion had particular valency or significance (either emotional or professional) for the supporters, for instance when it was part of a health promotion consultation,^18^ or a discussion about cancer, the supporters’ own views and interpretations of the materials appeared to take precedence.^46^ This might mean that supporters used the materials to emphasize normative behaviour regarding health promotion (rather than outlining choices) or avoided aspects of the resource they found difficult or upsetting. Relying on reports of carers or professionals, rather than using observational methods, perhaps gave a more positive view of the impact of accessible information in real‐life settings, such as visiting the doctor.^47^ A drawback for these papers is the very small numbers of people involved in the studies with ten or fewer participants with intellectual disabilities in all cases.

### 4.5 Group 5: Evaluation of effects of accessible information

These papers are from different disciplinary traditions (clinical psychology, psychiatry, speech and language therapy, education, computational linguistics) although all aimed to evaluate the actual use of adaptations to make information more accessible to people with intellectual disabilities using experimental paradigms under relatively controlled conditions. An institutional commitment to evidence‐based medicine^48^ was evidenced in the rationales authors offered for their research. Papers were published in peer‐reviewed journals aimed at a clinical academic audience. Authors compared the impact of adapted materials to information provided in conventional or more complex formats and used quantitative methodologies to compare between and within groups. A number of these papers used standardized tests of reading or comprehension to categorize participants with ID. The entrance of scholars working in linguistics^49^ and computational linguistics^50^, ^51^ into this area is noteworthy.

The adaptations to the materials that were offered to people with intellectual disabilities were various, and strategies included adding or substituting visual images such as photographs,^20^, ^52^ clipart,^51^ symbols^19^, ^53^ or pictures;^54^, ^55^ or changing the complexity, linguistic features or presentation of the text.^20^, ^50^, ^51^, ^56^ The paper by Feldman et al.^56^ was the only one to explore the impact of presenting text in an audio rather than video format. This variety does pose some difficulties in drawing overall conclusions from the studies’ findings.

These studies recruited larger groups of participants (between 13 and 85 people), although tended to do so “opportunistically.” Most were described as having “mild” or “moderate” intellectual disabilities and potential participants with very restricted or no verbal language were excluded.

Nevertheless, the impact of using accessible information was disappointing. Where understanding of the texts was assessed as an outcome measure, and adapted and non‐adapted texts were compared,^19^, ^20^, ^52^, ^53^, ^54^, ^55^, ^56^ only one of these studies^53^ found an advantage for groups who had been given information that had been simplified linguistically or otherwise adapted to make it easier to understand. On the whole, the cognitively more able, or those who did better on reading tests, seemed to do a better job of decoding. For those who struggled more, adapting the text in various ways did not tend to help.

## 5 SYNTHESIS

In this section, we identify higher order themes through reflecting on the conceptual, methodological and empirical differences between our groupings. These are represented as tensions between diverse positions with implications for practice, policy and future research; we reflect on these in the final discussion section.

### 5.1 Public or individualized resources?

Creators of accessible resources are faced with a dilemma regarding whether they address potential consumers of their texts as a group (whether they are seen as inadequate communicators or disabled by social barriers) or individuals. Much accessible information can be described as generic^57^ or public communication defined as large scale in distribution and received by a heterogenous audience. The relationship between the sender and receiver is asymmetrical (from expert to novice), impersonal and controlled by the sender.

Group 1 authors focused on producing accessible information as “public information” and Group 5 authors address its effectiveness on this level. Therefore, for Group 1, success can be measured in the uptake of the resource (eg, how many downloads from a website). For Group 5, the resource is judged on how far it assists the understanding of a presumably representative group of people with intellectual disabilities.

For others, particularly Group 4, the accessibility of a resource is determined by the extent to which it has been crafted to meet the individual requirements of the person with intellectual disabilities. This can be designed into an assessment process that precedes the creation of the resource^45^ or into the format of the resource itself, such as Talking Mats^58^ or Books Beyond Words^59^ which both present pictures or symbols only that are used and responded to on an individual level by the person with ID.

### 5.2 Literacy skills or literacy practices?

Use of reading assessments and readability metrics among Group 5 authors points to an underlying model of literacy as a set of cognitively based technical skills. Accessible information is proposed as a resource that demands fewer of these skills in decoding texts of people with intellectual disabilities. There are variations in research methodologies, but all involved assessment of individuals looking by themselves at texts chosen and presented by the researchers, in standardized conditions.

A different view of literacy is suggested by the authors who focused more on the use of accessible information in more naturalistic settings, where the texts were of greater personal relevance to the person with intellectual disabilities and those supporting them. These encounters can be seen as “literacy events”; a term common within New Literacy Studies (NLS)^60^ to describe “any occasion in which a piece of writing is integral to the nature of the participants’ interactions and their interpretative processes (p. 50) “.^61^ NLS researchers challenge (still) prevailing definitions of literacy as a set of decontextualized individual skills and reconceptualize it as social practices (underpinned by ideologically reinforced understandings of the nature of literacy and its uses and effects), often involving more than just the individual reader.

Group 4 authors reflect these sorts of concerns, examining how people with intellectual disabilities, supporters and accessible texts come together in processes of meaning making that relate to participants wider social goals. They offer a more nuanced view of the role of carers as “literacy mediators”^62^ than authors in the other groups, who promote the importance of giving support to people with intellectual disabilities in using accessible information as straightforwardly facilitative.

### 5.3 People with intellectual disabilities and professionals: whose expertise?

The different groups of studies adopted diverse positions on the role of people with intellectual disabilities in the initiation, design and evaluation of accessible information along a continuum of involvement.^63^ At one end of the continuum, judgments about the readability of a text were calculated through computer algorithms^50^—people with intellectual disabilities were not involved at all. In other Group 5 studies, people with intellectual disabilities were involved as research “subjects” usually recruited through service settings that they attended, where it might be argued they were something of a captive audience. As participants in these studies, people with intellectual disabilities did not always have significant involvement in the design of the materials they were given to test.

At the other end of the continuum of involvement, people with intellectual disabilities were described as the originators of ideas for accessible materials,^43^, ^64^ authors and editorial advisers,^35^, ^65^ or coresearchers^31^, ^34^ into the impact of accessible information.

Between these two positions, people with intellectual disabilities were engaged as consultants at the beginning and quality checkers and approvers towards the end of the design and production process. The importance of involving people with intellectual disabilities was stressed, but the expertise of health and communication professionals (often bringing together professionals from intellectual disability services, primary and secondary care and trust corporate departments) was taken as the starting point for resource design and refinement.

These different positions indicated a tension in the literature regarding the nature of relevant expertise when it comes to creating and evaluating accessible information. From the perspective of literature originating from self‐advocacy and disability studies scholarship, people with intellectual disabilities are regarded as “experts by experience”^66^ on accessible information, bringing experiential authority^67^ based on their location within and knowledge of communities of disabled people.

Professional expertise, on the other hand, is usually understood to transcend specific circumstances and life‐experiences. For the researchers, particularly in Group 1 who orientated most to institutional requirements, with a high value put on accountability, avoidance of risk and respect for bureaucratic hierarchies, expert professional knowledge was highlighted as an indicator of quality of accessible information.

## 6 DISCUSSION

### 6.1 Limitations of this study

Meta‐narrative review regards intuition, personal and professional knowledge and networks, and serendipity as resources available to reviewers, although at the expense of the replicability of the review. We therefore acknowledge that this review is itself a crafted narrative, albeit one that we have tried to support throughout with reference to our primary sources. Other reviewers might well identify different groupings of studies and highlight different themes.

The relatively discrete focus of our review and our limited resources in undertaking it meant adherence to the guiding principles of historicity (unfolding of research traditions over time) and peer review were a challenge. We refer throughout to “groupings” rather than “research traditions” as we found limited mutual awareness and intergroup citation, even between very similar studies, meaning that it was not easy to get a sense of later work building on and elaborating earlier work. Our engagement with peer review only extended as far as seeking feedback from peers and mentors; ideally, we would have undertaken the review with support from a reference group that included different stakeholders, including practitioners, policymakers and people with intellectual disabilities.

### 6.2 Summary of findings

We began this review with reference to the very extensive claims that have been made for the potential impact of accessible health information for people with intellectual disabilities and health providers, including clearer understanding of health and illness, increased decision making, self‐management, better health, consumer satisfaction with care and cost savings. From reviewing the literature, we were unable to find clear evidence that introducing accessible information leads to these outcomes, at least when it is disseminated as public information.

Perhaps such a conclusion is inevitable; after all, it is difficult to establish a clear causal link between delivery of written health information and changes in health behaviours in the wider population.^68^ There was no clear consensus among the papers reviewed as to what constituted the most important impacts of adapted health information, how to measure these or what would constitute an acceptable degree of quality and rigour in evaluation. There were a number of favourable reports on the reception and uptake of adapted health information by people with intellectual disabilities and those who support them. However, we are not yet able to conclude that a presumably basic aspect of adapted information, that it is reliably easier to understand than a non‐adapted version by people with intellectual disabilities, has been achieved.

The reviewed literature does suggest that adapted health information has a better chance of making an impact when it is tailored to an individual's individual requirements for information and communicative support (this is also true for the wider population^69^). We have also been alerted to the danger that inequal power relationships between people with intellectual disabilities and supporters/professionals can be reproduced in literacy events involving accessible information.

We noted some additional impacts not directly related to individual health outcomes; the process of creating and designing accessible health information may bring together different social groups in new ways that might well benefit people with intellectual disabilities. People with intellectual disabilities were able to access new forms of social capital as authors of accessible information and arbiters of its quality. We also found examples of specialist intellectual disability staff working more closely with mainstream staff to design and disseminate accessible information.

## 7 DIRECTIONS FOR FUTURE RESEARCH

In selecting our papers for review, we found that we had to omit literature that was potentially very relevant and valuable, because the authors had not explicitly included people with intellectual disabilities in their research samples, or had in some cases excluded them.^70^ In the wider literature relating to written health communication, simplification of health messages and health literacy we found widely cited review studies, that explored, for instance the usefulness of using visual images in health communication^71^ or compared different strategies for simplifying health information.^72^ We also discovered a separate literature on making information accessible for people experiencing aphasia after a stroke.^73^, ^74^ Further research is needed to clarify whether there are common strategies that are likely to improve the accessibility for a range of groups who need communicative support, or whether different groups have different requirements. Some of the authors of our reviewed papers suggested that resources developed for people with intellectual disabilities offer many benefits to the wider population. This claim needs further empirical exploration.

The review revealed a lack of information about how people with intellectual disabilities and the people with support them use accessible information in their everyday lives. Unfortunately, observational research on the communicative experiences of people with intellectual disabilities is still scarce^75^; this is even more the case regarding literacy practices.^76^, ^77^

If these resources are being used in real‐life situations, how can we monitor their impact and specify appropriate outcomes? Different quantitative and qualitative (including ethnographic and interactional) methodologies are needed to address the impact of accessible information. Biomedical research into health communication assumes that better communication will lead to higher rates of uptake of services and adherence to professional advice. However, it is also possible that greater understanding and engagement with health information (including understanding drawbacks and side‐effects of procedures and interventions) may lead to lower rates of take‐up.^78^

There was very little attention paid in any of the literature reviewed to the content of accessible information, beyond its readability. There was rarely a critique of the concepts articulated in the resources and the authority of professional knowledge and advice contained within them. In fact of all the literature we reviewed, only one paper^65^ discussed the importance of accessible information offering clear choices to people with intellectual disabilities, including the option to decline treatment or engagement in self‐care practices.

The information offered in the accessible texts—usually institutionally sanctioned information about biomedicine or services, was assumed to be both neutral and authoritative. The underlying theory of communication pervasive in the literature is based on the mechanistic sender–receiver model of Shannon & Weaver^79^ whereby information is a commodity passed from one person to another. Making information “accessible” therefore is equated with breaking down barriers to make sure that information flows freely, the expected direction of flow being from the more to the less knowledgeable.^22^

Alternative conceptualizations of how communication works from a post‐modernist and social constructionist perspective regard language itself as constitutive of social phenomena and identities. Culturally and historically, specific expectations of how people should behave are transmitted linguistically through the choice and coordination of elements of different modes of meaning (words, picture, gestures).^80^ Moreover, scholars from cultural and media studies challenge the idea that^81^ audiences simply absorb the message of the text, instead describing text recipients as involved in active meaning making, bringing their own resources to bear on interpretation of a text. This suggests a direction for future research that seeks to deconstruct accessible health information texts to discern how people with intellectual disabilities, health professionals and biomedical institutions are constructed within them and also map how texts are taken up, recontextualized and transformed in use.^82^

## CONFLICTS OF INTEREST

None.
